# Mott Transition in the Hubbard Model on Anisotropic Honeycomb Lattice with Implications for Strained Graphene: Gutzwiller Variational Study

**DOI:** 10.3390/ijms24021509

**Published:** 2023-01-12

**Authors:** Grzegorz Rut, Maciej Fidrysiak, Danuta Goc-Jagło, Adam Rycerz

**Affiliations:** 1 Institute for Theoretical Physics, Jagiellonian University, Łojasiewicza 11, PL-30348 Kraków, Poland; 2 Verisk Analytics Sp. z o.o., Rakowicka 7, PL–31511 Kraków, Poland

**Keywords:** graphene, electron correlations, Gutzwiller approximation

## Abstract

The modification of interatomic distances due to high pressure leads to exotic phenomena, including metallicity, superconductivity and magnetism, observed in materials not showing such properties in normal conditions. In two-dimensional crystals, such as graphene, atomic bond lengths can be modified by more than 10 percent by applying in-plane strain, i.e., without generating high pressure in the bulk. In this work, we study the strain-induced Mott transition on a honeycomb lattice by using computationally inexpensive techniques, including the Gutzwiller Wave Function (GWF) and different variants of Gutzwiller Approximation (GA), obtaining the lower and upper bounds for the critical Hubbard repulsion (*U*) of electrons. For uniaxial strain in the armchair direction, the band gap is absent, and electron correlations play a dominant role. A significant reduction in the critical Hubbard *U* is predicted. Model considerations are mapped onto the tight-binding Hamiltonian for monolayer graphene by the auxiliary Su–Schrieffer–Heeger model for acoustic phonons, assuming zero stress in the direction perpendicular to the strain applied. Our results suggest that graphene, although staying in the semimetallic phase even for extremely high uniaxial strains, may show measurable signatures of electron correlations, such as the band narrowing and the reduction in double occupancies.

## 1. Introduction

The Hubbard model, initially proposed to describe interaction-driven transition between conducting and insulating systems [1,2], needs to be carefully applied in low dimensions, where exact solutions (when available) [3,4] show substantially different ground-state properties than approximate solutions obtained using methods such as Hartree–Fock (HF) [2,5], GWF or Gutzwiller Approximation (GA) [6,7,8,9,10]. For this reason, computationally-expensive numerical techniques, such as Quantum Monte Carlo (QMC) [11] or a more recent tensor-network method [12,13,14], are usually employed for the Hubbard model in two dimensions, for which an exact solution is missing.

A notable exception, however, is a honeycomb lattice, for which relatively simple techniques, including GWF [15] or CPA [16,17], provide reasonable approximations for the critical Hubbard interaction, differing from the QMC value, $U_{c}=3.86\left(1\right)\;t_{0}$ [18] (with $t_{0}$ being the nearest-neighbor hopping integral and the number in parenthesis denoting uncertainty for the last digit), by less than 10%. For a comparison, the HF method gives $U_{c}^{\left(\mathrm{HF}\right)}=2.23\;t_{0}$ [19] for the same lattice.

Since the advent of graphene [20,21], half-filled, fermionic honeycomb-lattice systems have attracted renewed attention, as they emulate several field-theoretical phenomena in condensed matter [22]. The effective Hubbard model for monolayer graphene with on-site interaction $U_{\mathrm{eff}}\approx 1.6\;t_{0}$ was proposed [23], suggesting that large isotropic strain may drive this system from semimetallic towards the Mott-insulating phase [24,25] in analogy with high pressure changing properties of various bulk materials [26,27,28,29,30]. The effects of electron correlations are usually more pronounced in graphene nanosystems, where quantum fluctuations are reduced and magnetic moments may form near free edges [31,32,33] (although defining metallic and insulating states for a nanosystem is more cumbersome than for a bulk system [34,35]). We further notice that artificial graphene-like systems allow one to tune the interaction in a wider range than actual graphene [36,37,38,39]. Yet another possibility to study electron correlations has opened with the fabrication of twisted bilayer graphene [40,41].

A separate issue concerns the bandgap opening due to the spatial rearrangement of atoms in strained graphene (a so-called two-dimensional Peierls instability), which may turn the system into an insulator before the Mott transition occurs [42,43,44,45,46]. On the contrary, a weak electron–phonon interaction of the Holstein type, which may appear in graphene on some substrates, is predicted to favor the semimetallic phase [47].

**Figure 1:**
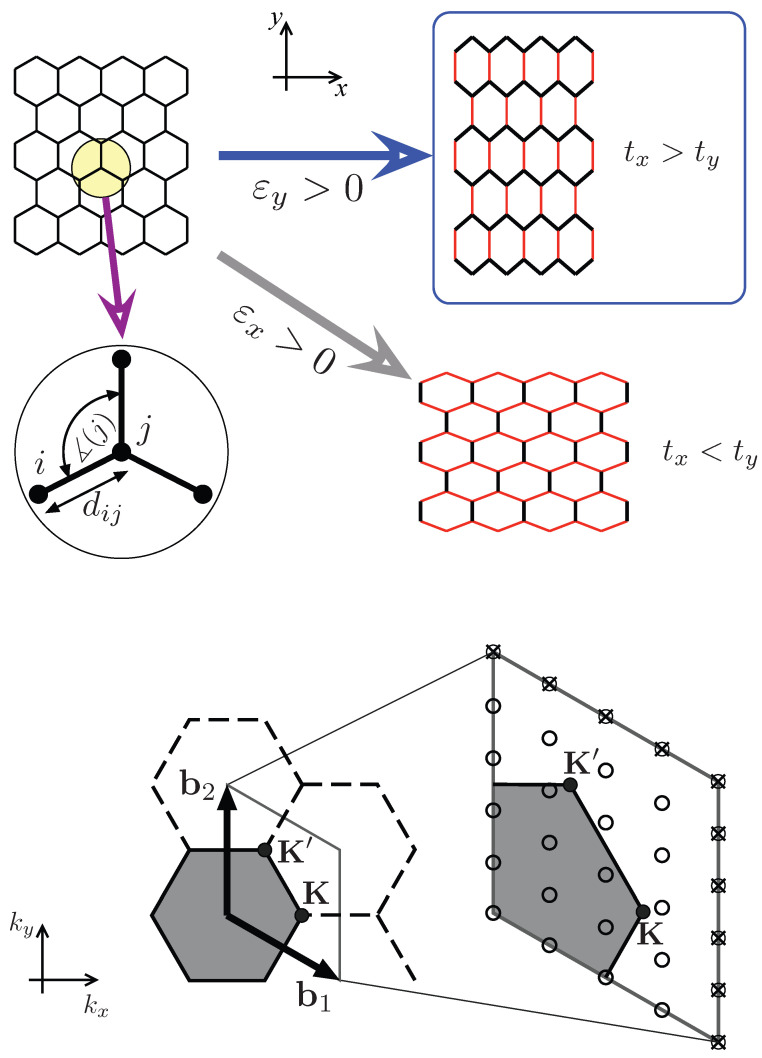
**Top**: Honeycomb lattice subjected to uniaxial strain in selected direction (see the coordinate system). Zoom-in visualizes the distance (bond length) $d_{ij}$ between atoms *i* and *j* and in-plane angle with the vertex at site *j* (∡(j)). **Bottom**: Hexagonal first Brillouin zone (FBZ) of the reciprocal lattice, with (dimensionless) basis vectors $b_{1}=\left(2\pi /\sqrt{3}\right)\;\left(\sqrt{3},-1\right)$ and $b_{2}=\left(2\pi /\sqrt{3}\right)\;\left(0,2\right)$, and the symmetry points, K=(4π/3,0) and $K^{\prime }=\left(2\pi /3,2\pi /\sqrt{3}\right)$ coinciding with Dirac points in the absence of strain. The magnified area shows discretized FBZ for a finite system of $N=2N_{x}N_{y}$ atoms with periodic boundary conditions [see Equation (6)]. (The values of $N_{x}=4$ and $N_{y}=5$ are used for illustration only).

In this paper, the discussion is limited to a honeycomb lattice strained along main crystallographic axes (see Figure 1) supposing that the bipartite structure of the lattice is preserved under strain. In turn, there are two different values of the nearest-neighbor hopping integral in a single-particle Hamiltonian, $t_{x}$ and $t_{y}$, corresponding to electron hopping along the zigzag direction ($t_{x}$) or along the armchair direction ($t_{y}$). To obtain a direct mapping between the strain applied and the hopping integrals, a version of the Su–Schrieffer–Heeger (SSH) model [48] is developed, with microscopic parameters adjusted to match elastic properties of graphene [49]. Once fixed strain is applied in a selected direction, the lattice is allowed to relax along the perpendicular direction to reach a conditional energy minimum (a zero perpendicular stress case). We further focus our attention on the strain applied along the armchair direction, for which the system evolves towards a collection of weakly-coupled one dimensional chains [50,51,52], allowing one to expect that, once the effective Hubbard model is considered, the Mott transition may appear for smaller values of $U_{\mathrm{eff}}$ than for isotropic strain.

The remaining part of the paper is organized as follows. In Section 2, we briefly present approximate approaches to the effective Hubbard Hamiltonian (including HF, GWF, and GA). Moreover, in Section 2, we show some original data, illustrating how these approaches work for anisotropic honeycomb lattice. In Section 3, we discuss our numerical results concerning the phase diagram of the effective Hubbard model with arbitrary parameters ($t_{x}⩾t_{y}$ and $U_{\mathrm{eff}}$), the evolution of the model parameters in graphene subjected to uniaxial strain, and approximate formula relating the reduction in $U_{c}$ to strain-induced anisotropy of the Fermi velocity. The effects of electron correlations on selected measurable quantities are also presented in Section 3. The concluding remarks are given in Section 4.

Next to the main text, in Appendix A, the Coherent Potential Approximation (CPA) is briefly described. In Appendix B, we present the auxiliary SSH model proposed to relate the physical strain onto the microscopic parameters of the effective Hubbard model.

## 2. Model and Methods

### 2.1. The Anisotropic Hubbard Model

Our analysis of electron correlations on the anisotropic honeycomb lattice starts from the Hamiltonian

(1) $$H=\sum_{\langle ij\rangle ,s}t_{ij}\left(c_{i,s}^{†}c_{j,s}+H.c.\right)+U\sum_{j}n_{j↑}n_{j↓},$$

with the first sum running over pairs of nearest-neighbors 〈ij〉 and spin up/down orientations (s=↑,↓), and the hopping-matrix elements are given by

(2) $$t_{ij}=\left\{\begin{array}{cc} -t_{x} & \mathrm{if}\;i,j\;\mathrm{belongs to same zigzag line},\\ -t_{y} & \mathrm{otherwise}. \end{array}\right.$$

(Without loss of generality, we suppose the coordinate system is oriented as depicted in Figure 1) The remaining symbols in Equation (1) are a creation (annihilation) operator for electron with spin *s* on the lattice site *i*, $c_{i,s}^{†}$ ($c_{i,s}$), $n_{is}=c_{i,s}^{†}c_{i,s}$, and the on-site Hubbard repulsion *U*. We further limit our considerations to the ground state and suppose the half-filling, i.e., one electron per lattice site, $\bar{n}=\langle n_{i↑}+n_{i↓}\rangle =1$.

In principle, the ground-state properties of the model defined by Equations (1) and (2) can be discussed as functions of two dimensionless parameters, e.g., $t_{y}/t_{x}$ and $U/t_{x}$. The relation between parameters $t_{x}$ and $t_{y}$ and strain applied to graphene is discussed later in this section. However, first, we briefly present approximate approaches capable to distinguish whether ground state of the Hamiltonian (1) is semimetallic or insulating.

### 2.2. Hartree–Fock Approximation

**Figure 2:**
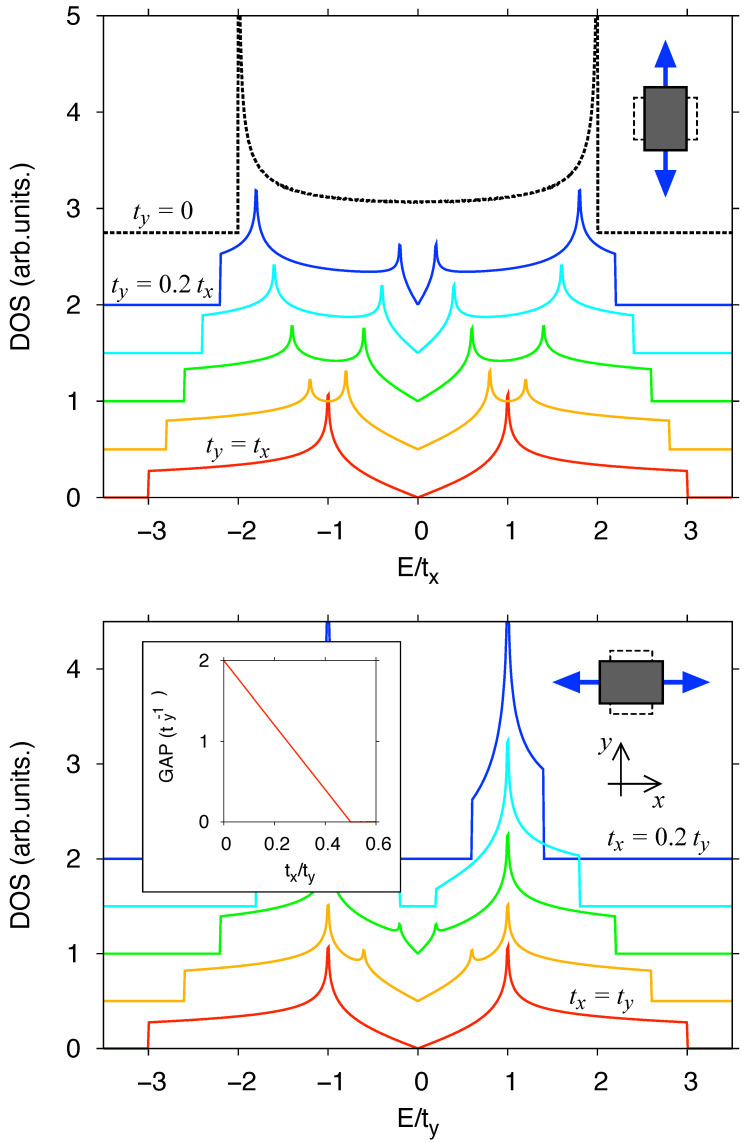
Density of states for the Hamiltonian (1) with U=0 displayed as a function of energy. Top: strain applied in the armchair direction ($t_{y}⩽t_{x}$). Bottom: strain applied in the zigzag direction ($t_{y}⩾t_{x}$). The ratio $t_{<}/t_{>}$ [with $t_{<}=\min\;\left(t_{x},t_{y}\right)$ and $t_{>}=\max\;\left(t_{x},t_{y}\right)$] is varied between the lines with the steps of 0.2. A vertical offset is applied to each dataset except from the isotropic case ($t_{x}=t_{y}$). Inset shows the band gap, appearing for $t_{x}<0.5\;t_{y}$ due to the Peierls transition.

Although a honeycomb lattice is bipartite and the antiferromagnetic order is possible, its peculiar band structure suppresses antiferromagnetism at small *U* [15]. Since a single particle density of states (i.e, density of states at U=0) is linear for low energies, see Figure 2, there is no Fermi surface that could produce magnetic instability also for small U>0.

Within the Hartree–Fock approximation, the interaction part in the Hamiltonian (1) is replaced by

(3) $$U\hat{D}\overset{\mathrm{HF}}{=}U\sum_{i}\left(\langle n_{i↑}\rangle n_{i↓}+n_{i↑}\langle n_{i↓}\rangle -\langle n_{i↑}\rangle \langle n_{i↓}\rangle \right),$$

where we have introduced the operator $\hat{D}=\sum_{j}n_{j↑}n_{j↓}$ measuring the number of double occupancies. We further impose the antiferromagnetic order,

(4) $$\langle n_{i↑}\rangle =\frac{\bar{n}+\lambda_{i}m}{2},\;\;\;\;\;\;\langle n_{i↓}\rangle =\frac{\bar{n}-\lambda_{i}m}{2},$$

where $\lambda_{i}=1$ if *i* belongs to one sublattice (*A*), or $\lambda_{i}=-1$ if *i* belongs to the other sublattice (*B*), and *m* is the magnetization ($\left|m\right|⩽\bar{n}$), and the half filling ($\bar{n}=1$). The above yields the HF ground-state energy per site

(5) $$\begin{array}{cc} \frac{E_{G}^{\left(\mathrm{HF}\right)}}{N} & =-\frac{2}{N}\sum_{k}\sqrt{E_{k}^{2}+{\left(\frac{Um}{2}\right)}^{2}}+\frac{U(1\;+\;m^{2})}{4}, \end{array}$$

where the factor 2 accounts for s=↑,↓, and the summation runs over quasimomenta $k\equiv (k_{x},k_{y})$ in the first Brillouin zone, namely

(6) $$\begin{array}{cc} k_{x} & =\frac{2\pi }{N_{x}}n_{x},\;\;\;\;\;\;k_{y}=\frac{4\pi }{\sqrt{3}}\left(\frac{n_{y}}{N_{y}}-\frac{n_{x}}{2N_{x}}\right),\\ n_{x} & =0,1,\cdots ,N_{x}\;-\;1,\;\;\;\;n_{y}=0,1,\cdots ,N_{y}\;-\;1, \end{array}$$

with $N_{x,y}$ being the number of unit cells in x,y direction, $N=2N_{x}N_{y}$ (the periodic boundary conditions are imposed). For a sufficiently large number of points in the momentum space, say $N_{x},N_{y}≳10^{3}$, one can usually works with a square inverse lattice (omitting the term $\propto n_{x}$ in the expression for $k_{y}$) [53]; nevertheless, the discretization of $\left(k_{x},k_{y}\right)$ as given in Equation (6) becomes crucial when discussing the finite-size effects for small *N*. The single-particle energies for anisotropic honeycomb lattice are given by

(7) $$E_{k}=t_{x}\sqrt{a_{k}^{2}+b_{k}^{2}},$$

with

(8) $$\begin{array}{cc} a_{k}= & -\cos\left(\frac{k_{x}}{2}+\frac{\sqrt{3}k_{y}}{2}\right)-\cos\left(\frac{k_{x}}{2}-\frac{\sqrt{3}k_{y}}{2}\right)-\frac{t_{y}}{t_{x}},\\ b_{k}= & \sin\left(\frac{k_{x}}{2}+\frac{\sqrt{3}k_{y}}{2}\right)-\sin\left(\frac{k_{x}}{2}-\frac{\sqrt{3}k_{y}}{2}\right). \end{array}$$

Next, the density of states is defined as

(9) $$\rho \left(E\right)=2N^{-1}\sum_{k}\left[\delta \left(E-E_{k}\right)+\delta \left(E+E_{k}\right)\right],$$

with two parts corresponding to the conduction (E>0) and valence (E<0) band, and satisfying the normalization conditions: $\int_{-\infty }^{0}dE\;\rho \left(E\right)=\int_{0}^{\infty }dE\;\rho \left(E\right)=1$. If m≠0, the minimization of $E_{G}^{\left(\mathrm{HF}\right)}$ given by Equation (5) brought us to

(10) $$1=\int_{E<0}dE\;\rho \left(E\right)\frac{U/2}{\sqrt{E^{2}+{\left(Um/2\right)}^{2}}}.$$

In case the solution of Equation (10) does not exist, the minimum of $E_{G}^{\left(\mathrm{HF}\right)}$ corresponds to m=0.

Unlike for square lattice, for which one obtains m≠0 for any U>0 [5], on a honeycomb lattice the minimization gives m=0 for $U⩽U_{c}^{\left(\mathrm{HF}\right)}$ and m≠0 for $U>U_{c}^{\left(\mathrm{HF}\right)}$ [15]. This can be easily understood in the case of unstrained (or uniformly strained) lattice, for which $t_{x}=t_{y}=t_{0}$ and the density of states can be approximated by

(11) $$\rho \left(E\right)\approx \rho_{\Lambda }\left(E\right)=\left\{\begin{array}{cc} \frac{2}{\Lambda {}^{2}}\left|E\right| & \mathrm{for}\;\;|E|⩽\Lambda ,\\ 0 & \mathrm{for}\;\;|E|>\Lambda , \end{array}\right.$$

with a cut-off energy of $\Lambda ={\left(\sqrt{3}\pi \right)}^{1/2}\;t_{0}≃2.33268\;t_{0}$. The above is equivalent, for |E|⩽Λ, to $\rho_{\Lambda }\left(E\right)=2A\left|E\right|/\left[N\pi {\left(\hbar v_{F}\right)}^{2}\right]$, with A being the system area, $v_{F}=\frac{1}{2}\sqrt{3}\;at_{0}/\hbar$ the Fermi velocity, and *a* the lattice parameter [54]. It is straightforward to show that m≠0 appears above $U_{c}^{\left(\Lambda \right)}=\Lambda$, being not far from the value reported in Ref. [19].

The values of $U_{c}^{\left(\mathrm{HF}\right)}$ following from numerical minimization of $E_{G}^{\left(\mathrm{HF}\right)}$ given by Equation (5) for the actual density of states are presented in Section 3.

### 2.3. Gutzwiller Wavefunction

Generalized Gutzwiller wavefunction, allowing antiferromagnetic order, was applied in Ref. [15] to find out that correlated but paramagnetic solution remains stable up to the region of the Mott semimetal-insulator transition. Although several features of the solution are altered when employing more advanced techniques [18], the values of $U_{c}$ following from GWF are surprisingly close to those obtained within large-scale computer simulations for a honeycomb lattice. Investigating the variational wavefunction

(12) $$|\Psi_{\mathrm{GWF}}\rangle =e^{-\eta \hat{D}}|\psi_{0}\left(m\right)\rangle ,$$

where $|\psi_{0}\left(m\right)\rangle$ denotes a Slater determinant corresponding to a given magnetization in Equation (5) and η is another variational parameter (quantifying the role of electron correlations), one needs to minimize the ground-state energy

(13) $$E_{G}^{\left(\mathrm{GWF}\right)}=\frac{\langle \psi_{0}\left(m\right)|e^{-\eta \hat{D}}He^{-\eta \hat{D}}|\psi_{0}\left(m\right)\rangle }{\langle \psi_{0}\left(m\right)|e^{-2\eta \hat{D}}|\psi_{0}\left(m\right)\rangle },$$

with respect to η and *m*. In many cases, the system may prefer to reduce *m* (even to m=0) and increase η, allowing to expect that, in general, $U_{c}^{\left(\mathrm{GWF}\right)}⩾U_{c}^{\left(\mathrm{HF}\right)}$.

**Figure 3:**
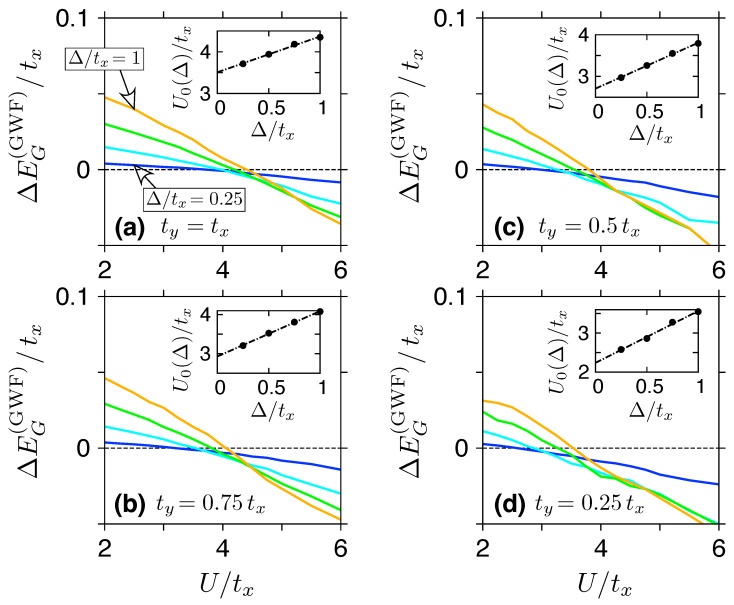
(**a**–**d**) Main: Energy difference between the antiferromagnetic Gutzwiller variational energy $E_{G}^{\left(\mathrm{GWF}\right)}\left(m\right)$ [see Equation (13)] and the paramagnetic solution $E_{G}^{\left(\mathrm{GWF}\right)}\left(0\right)$ obtained from VMC simulations as a function the on-site Hubbard repulsion (*U*). The parameter η is optimized for a fixed m=Δ/U (or m=0); Δ is varied between the lines from $\Delta /t_{x}=0.25$ to $\Delta /t_{x}=1$, with the steps of 0.25. The hopping anisotropy $t_{y}/t_{x}$ is varied between the panels. Inset shows the value of $U=U_{0}\left(\Delta \right)$ at which $\Delta E_{G}^{\left(\mathrm{GWF}\right)}$ changes sign for a given Δ. The extrapolation to Δ→0 yields the critical values of $U_{c}^{\left(\mathrm{GWF}\right)}$ given in Table 1. (Statistical errorbars are too small to be shown on the plots).

Several approximated techniques for calculating the averages in Equation (13) were developed [7,8,9,10,15]. Here, we apply Variational Monte Carlo (VMC), described in detail in Ref. [10]. To determine the value of $U_{c}^{\left(\mathrm{GWF}\right)}$, we have directly followed the procedure proposed by Martelo et al. [15]. For a fixed value of the gap (Δ≡Um), the energy difference $\Delta E_{G}^{\left(\mathrm{GWF}\right)}=E_{G}^{\left(\mathrm{GWF}\right)}\left(m\right)-E_{G}^{\left(\mathrm{GWF}\right)}\left(0\right)$, where the parameter η is optimized independently for m=0 and m≠0, changes sign at some $U=U_{0}\left(\Delta \right)$. Numerical extrapolation of $U_{0}\left(\Delta \right)$ with Δ→0 allows one to determine the critical value of $U_{c}^{\left(\mathrm{GWF}\right)}$. Selected examples, for $t_{y}⩽t_{x}$ (i.e., strain applied in the armchair direction) and the system size of N=200 sites ($N_{x}=N_{y}=10$), are presented in Figure 3. For more details of the simulation, see Ref. [55].

### 2.4. Gutzwiller Approximation and Its Variants

To efficiently study the effects of electron correlations present in $|\Psi_{\mathrm{GWF}}\rangle$, see Equation (12), one can also adopt the Gutzwiller Approximation (GA) and find out how the number of double occupancies is reduced comparing to the HF solution $|\psi_{0}\left(m\right)\rangle$. Within GA, which is exact in the infinite dimension limit, the correlation functions ${\langle c_{is}^{†}c_{js}\rangle }_{\mathrm{GWF}}=\langle \Psi_{\mathrm{GWF}}|c_{is}^{†}c_{js}|\Psi_{\mathrm{GWF}}\rangle /\langle \Psi_{\mathrm{GWF}}|\Psi_{\mathrm{GWF}}\rangle$ are approximated by

(14) $${\langle c_{is}^{†}c_{js}\rangle }_{\mathrm{GWF}}\overset{\mathrm{GA}}{=}q\left(\left\{\rho_{ll^{\prime }s^{\prime }}^{\left(0\right)}\right\};\left\{d_{l}\right\}\right)\;{\langle c_{is}^{†}c_{js}\rangle }_{0}$$

where the band-narrowing factor $q(\left\{\rho_{ll^{\prime }s^{\prime }}^{\left(0\right)}\right\};\left\{d_{l}\right\})$ depends only on the single-particle density matrix elements $\rho_{ll^{\prime }s^{\prime }}^{\left(0\right)}={\langle c_{ls^{\prime }}^{†}c_{l^{\prime }s^{\prime }}\rangle }_{0}$ with $l,l^{\prime }=i,j$ and $s^{\prime }=↑,↓$ (here, ${\langle \cdots \rangle }_{0}$ is the expectation value over the uncorrelated state; i.e., a single Slater determinant such as $|\psi_{0}\left(m\right)\rangle$), and $d_{l}={\langle n_{l↑}n_{l↓}\rangle }_{\mathrm{GWF}}$ (l=i,j) being the average double occupancies. The $\left\{d_{i}\right\}$ variables are further regarded as variational parameters to be determined by minimizing the Gutzwiller energy functional,

(15) $$E_{G}^{\left(\mathrm{GA}\right)}=2\sum_{\langle ij\rangle ,s}q\left(\left\{\rho_{ll^{\prime }s^{\prime }}^{\left(0\right)}\right\};\left\{d_{l}\right\}\right)\;t_{ij}\rho_{ijs}^{\left(0\right)}+U\sum_{j}d_{j}.$$

Several forms of the band-narrowing factor $q(\left\{\rho_{ll^{\prime }s}^{\left(0\right)}\right\};\left\{d_{l}\right\})$, being equivalent in the infinite dimension limit but producing slightly different results when applied to the system of a finite dimensionality, are used in the literature [8,56,57,58,59,60,61,62]. For the diagonal elements $\rho_{iis}^{\left(0\right)}={\langle n_{is}\rangle }_{0}$ parametrized as in Equation (4) with $\bar{n}=1$, one can impose $d_{i}\equiv d$ for all sites and rewrite the expression given in Ref. [61] as

(16) $$\begin{array}{cc} q(\left\{\rho_{ll^{\prime }s}^{\left(0\right)}\right\};\left\{d_{l}\right\}) & \equiv q\left(m,d\right)=\frac{4d}{1\;-\;m^{2}}\left[1\;-\;2d+\sqrt{{\left(1\;-\;2d\right)}^{2}-m^{2}}\right]. \end{array}$$

The variable *d* is bounded as $0⩽d⩽\frac{1}{4}\left(1\;-\;m^{2}\right)$, with the upper limit corresponding to the average double occupancy in the uncorrelated state $|\psi_{0}\left(m\right)\rangle$). The kinetic energy term can be estimated by referring to the Hartree–Fock energy $E_{G}^{\left(\mathrm{HF}\right)}$, see Equation (5), as $2\sum_{\langle ij\rangle ,s}t_{ij}\rho_{ij}^{\left(0\right)}=E_{G}^{\left(\mathrm{HF}\right)}-\frac{N}{4}U\left(1-m^{2}\right)$ even for *m* being away from the minimum of $E_{G}^{\left(\mathrm{HF}\right)}$. This brought us to

(17) $$\begin{array}{cc} \frac{E_{G}^{\left(\mathrm{GA}\right)}}{N} & =q\left(m,d\right)\times \left[-\frac{2}{N}\sum_{k}\sqrt{E_{k}^{2}+{\left(\frac{Um}{2}\right)}^{2}}+\frac{Um^{2}}{2}\right]+Ud. \end{array}$$

Numerical minimization of $E_{G}^{\left(\mathrm{GA}\right)}$, with respect to (m,d), truncates the optimization of both the density matrix $\left\{\rho_{i,j}^{\left(0\right)}\right\}$ and the parameters $\left\{d_{i}\right\}$. For the linear density of states $\rho_{\Lambda }\left(E\right)$, see Equation (11), one can easily find closed-from expression for $E_{G}^{\left(\mathrm{GA}\right)}$; the minima corresponding to m≠0 appear for $U>U_{c}^{\left(\Lambda ,\mathrm{GA}\right)}=1.270\;\Lambda =2.963\;t_{0}$, with the critical value lying between HF [19] and QMC [18] results for isotropic honeycomb lattice.

A slightly more accurate (but also more computationally expensive) approach can be constituted by parametrizing the uncorrelated state $|\psi_{0}\rangle$ not only via the magnetization *m*, as in the above, but via *all* independent parameters of the density matrix $\left\{\rho_{i,j}^{\left(0\right)}\right\}$. In particular, the auxiliary single-particle Hamiltonian determining $\left\{\rho_{i,j}^{\left(0\right)}\right\}$ contains the renormalized hopping integrals (${\tilde{t}}_{x}$ and ${\tilde{t}}_{y}$) which may differ from $t_{x}$ and $t_{y}$ in the multiparticle Hamiltonian (1). The resulting method, called the *Statistically-consistent Gutzwiller Approximation* (SGA), is presented in detail in Ref. [58].

Both (S)GA and GWF methods can be regarded as improvements to the mean-field (HF) solution, including some classes of quantum fluctuations. Since not all fluctuations are included, the AF order is artificially favored when searching for the energy minimum, and therefore, these methods usually underestimate the value of $U_{c}$. In order to bound $U_{c}$ from the top, we employ the scheme proposed by Martelo et al. [15], in which two solutions are compared: The paramagnetic GA solution, corresponding m=0 in Equation (17), with a complementary variational wavefunction,

(18) $$|\Psi_{B}\rangle =e^{-\kappa \hat{T}}|\Psi_{U\to \infty }\rangle ,$$

where κ is a variational parameter, $\hat{T}=\sum_{\langle ij\rangle ,s}t_{ij}\left(c_{is}^{†}c_{js}+\mathrm{H.c.}\right)$ is the kinetic-energy part of the Hamiltonian (1), and $|\Psi_{U\to \infty }\rangle$ is the ground state for U→∞. The critical value $U_{c}$ is than that estimated by finding a crossing point of $E_{G}^{\left(\mathrm{GA}\right)}$, Equation (17), with a fixed m=0 and optimized *d*, and the variational energy $E_{B}$ corresponding to $|\Psi_{B}\rangle$, Equation (18), with optimized κ.

For m=0, the factor q(m,d), Equation (16), reduces to a quadratic function of *d* and the functional $E_{G}^{\left(\mathrm{GA}\right)}$, Equation (17), reaches the minimum at $d=\frac{1}{4}\max\left[0,1-U/(8|\epsilon_{0}|)\right]$, leading to a form originally derived by Gutzwiller [63,64]

(19) $$E_{G}^{\left(\mathrm{GA}\right)}\left(m\;=\;0\right)=\left\{\begin{array}{cc} \epsilon_{0}+\frac{U}{4}-\frac{U^{2}}{64|\epsilon_{0}|}\; & \mathrm{for}\;U⩽8|\epsilon_{0}|,\\ 0 & \mathrm{otherwise}. \end{array}\right.$$

The symbol $\epsilon_{0}$ is the kinetic energy per site for U=0, namely $\epsilon_{0}=\int_{E<0}dE\;\rho \left(E\right)E$ [for the definition of ρ(E), see Equation (9)], taking the numerical value of $\epsilon_{0}/t_{x}=-1.57460$ for $t_{y}/t_{x}=1$, $\epsilon_{0}/t_{x}=-1.45540$ for $t_{y}/t_{x}=0.75$, $\epsilon_{0}/t_{x}=-1.36218$ for $t_{y}/t_{x}=0.5$, or $\epsilon_{0}/t_{x}=-1.29891$ for $t_{y}/t_{x}=0.25$.

**Figure 4:**
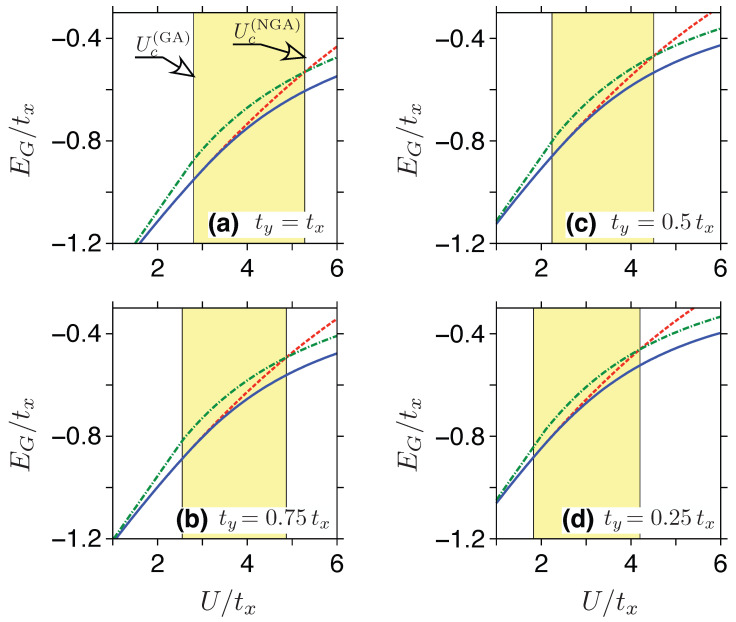
(**a**–**d**) The lower and the upper bounds to the critical Hubbard repulsion $U_{c}$ for anisotropic honeycomb lattice estimated by comparing different versions of the Gutzwiller Approximation described in the text. The lower bound ($U_{c}^{\left(\mathrm{GA}\right)}$) coincides with the splitting of the Gutzwiller energy for paramagnetic state, $E_{G}^{\left(\mathrm{GA}\right)}\left(m\;=\;0\right)$, given by Equation (19) [red dashed line] and the variational energy $E_{G}^{\left(\mathrm{GA}\right)}$, see Equation (17), with the parameters (m,d) optimized numerically [blue solid line], both displayed as functions of *U*. The value of $U_{c}^{\left(\mathrm{GA}\right)}$ is obtained via the extrapolation with m→0, similarly as for the VMC results in Figure 3. The intersection of $E_{G}^{\left(\mathrm{GA}\right)}\left(m\;=\;0\right)$ with $E_{G}^{\mathrm{NGA}}$, see Equation (20) [green dashed-dotted line] yields the upper bound ($U_{c}^{\left(\mathrm{NGA}\right)}$). The value of the $t_{y}/t_{x}$ ratio is varied between the panels. [For the numerical values of $U_{c}^{\left(\mathrm{GA}\right)}$ and $U_{c}^{\left(\mathrm{NGA}\right)}$, see Table 1].

In the limit of infinite dimensions, the variational energy $E_{B}$ associated with the state $|\Psi_{B}\rangle$ can be evaluated exactly, since the ground state $|\Psi_{U\to \infty }\rangle$ is the Néel antiferromagnet [65]. The variational energy reads

(20) $$\frac{E_{B}}{N}=\epsilon_{\mathrm{kin}}+\frac{U(1\;-\;m^{2})}{4}\equiv \frac{E_{G}^{\left(\mathrm{NGA}\right)}}{N}$$

where

(21) $$\begin{array}{cc} \epsilon_{\mathrm{kin}} & =\int_{E<0}dE\;\rho \left(E\right)E\left[-\tanh(2\kappa E)\right], \end{array}$$

(22) $$\begin{array}{cc} m & =\int_{E<0}dE\;\frac{\rho (E)}{\cosh(2\kappa E)}, \end{array}$$

are the kinetic energy per site and the sublattice magnetization (respectively). The so-called Néel–Gutzwiller Approximation (NGA) is constituted by substituting the density of states given by Equation (9) into Equations (21) and (22), and the subsequent minimization of $E_{B}\equiv E_{G}^{\left(\mathrm{NGA}\right)}$ with respect to κ. Selected numerical results, for $t_{y}⩽t_{x}$, are presented in Figure 4.

It is worth mentioning that (S)GA can be systematically improved by approaching the GWF solution, including consecutive corrections following from the relevant diagrammatic expansion [8,60,62] (we further notice that the detailed scheme for a honeycomb lattice is missing so far). A similar approach for $|\Psi_{B}\rangle$ is difficult due to the necessity of determining the ground state of the Heisenberg model ($|\Psi_{U\to \infty }\rangle$) as a first.

The selected numerical values of $U_{c}$, following on from the methods described in this section, are compared in Table 1.

A substantially different approach, the *Coherent Potential Approximation* (CPA), in which one considers random scattering of electrons with a given spin on motionless electrons with the opposite spin (instead of imposing some spin order), is described in Appendix A.

## 3. Results and Discussion

### 3.1. Phase Diagram

**Figure 5:**
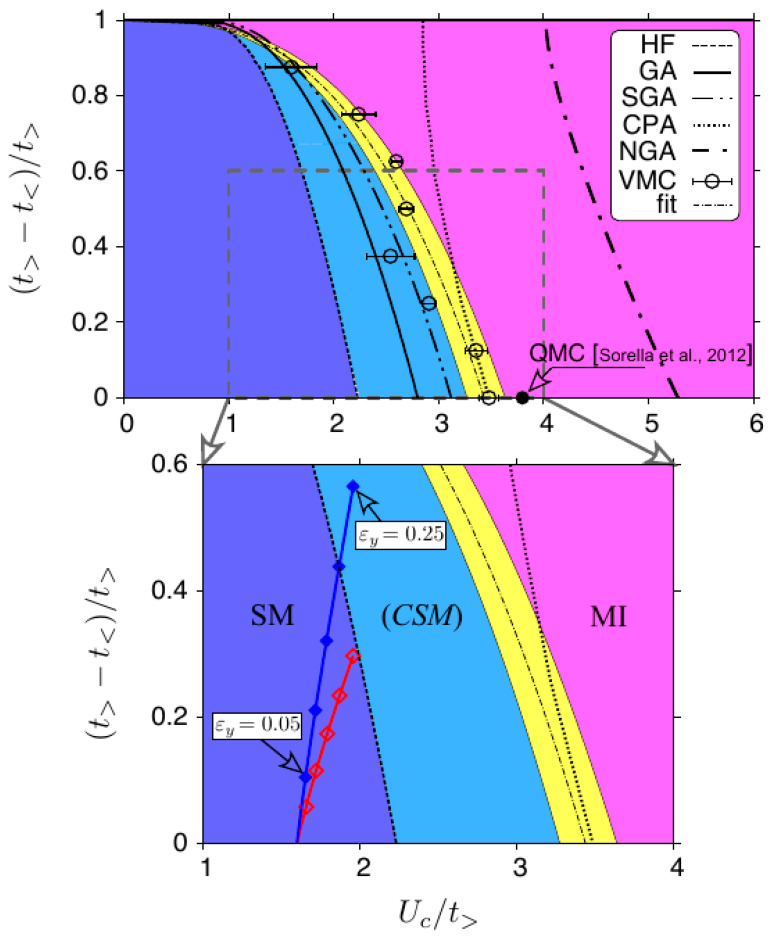
**Top**: Phase diagram for the Hubbard model on anisotropic honeycomb lattice, see Equations (1) and (2), with $t_{y}⩽t_{x}$ corresponding a gapless single-particle spectrum, see Figure 2. [Here, $t_{>}=t_{x}$ and $t_{<}=t_{y}$.] Lines depict the critical Hubbard repulsion estimated within the Hartree–Fock method [short dashed], Gutzwiller Approximation [thick solid], Statistically Consistent GA [long dashed-double dotted], Coherent Potential Approximation [dotted], and the Néel-state GA [long dashed-dotted]. Datapoints with errorbars are obtained from VMC simulations for the Gutzwiller Wave Function, see Equation (12); thin dash-dotted line represents a power-law fit given by Equation (23) with thin solid lines bounding the statistical uncertainty (yellow area). Quantum Monte Carlo value for the isotropic case, $U_{c}/t_{0}=3.86$ [18], is also marked (full circle). **Bottom**: A zoom in, with trajectories following from the SSH model for strained graphene (see Appendix B) for β=2 (red line/open symbols) and β=3 (blue line/closed symbols). Different datapoints for each value of β correspond to the applied strain $\varepsilon_{y}$ varied from $\varepsilon_{y}=0.05$ to 0.25 with the steps of 0.05. (GA and VMC results are omitted for clarity.) Remaining Labels/colored areas: the semimetallic phase (SM) [blue] with the correlated-semimetal range (*CSM*) [light blue] and the Mott insulator (MI) [magenta].

Our central results are presented in Figure 5, where we display the phase diagram for the Hamiltonian (1) with the strain applied in the armchair direction ($t_{y}⩽t_{x}$). A single-particle spectrum is gapless in such a case (see also Figure 2) since the positions of Dirac cones do not merge [66]; therefore, the metal-insulator (if it occurs) must be driven by electron–electron interaction. Most of the methods which we have presented in Section 2, i.e., HF, GA, and NGA, share a common feature that they allow one to take the limit of N→∞ numerically, and the results are free of finite-size (and statistical) errors. The same applies to SGA (see Ref. [58]) and CPA described in Appendix A. The case of GWF is different since VMC simulations produced considerable statistical errorbars (a triple standard deviation is marked for each datapoint) and may be biased due to possible systematic errors following from a limited system size of N=200 ($N_{x}=N_{y}=10$).

Despite the limited accuracy of VMC simulation results, they typically lie between the GA and CPA values (up to the errorbars), allowing us to regard the last two methods as providing approximate lower (GA) and upper (CPA) bounds to the value of $U_{c}$. However, it must be noticed that the ‘exact’ numerical value of $U_{c}^{\left(\mathrm{QMC}\right)}=3.86\;t_{0}$ of Ref. [18] (available only for the isotropic case, $t_{x}=t_{y}=t_{0}$) significantly exceeds $U_{c}^{\left(\mathrm{CPA}\right)}=3.49\;t_{0}$, and therefore the CPA results cannot be considered as upper bound to $U_{c}$ in a rigorous manner. When searching for a computationally-inexpensive technique providing a safe upper bound to $U_{c}$, one should rather refer to Neél-state Gutzwiller Approximation (NGA).

The relation between SGA and the above-mentioned methods is more complex, since more variational parameters defining the single-particle state $|\psi_{0}\rangle$ are optimized. In brief, the SGA ground-state energy is lower or equal to that obtained from GA, leading to $U_{c}^{\left(\mathrm{SGA}\right)}⩾U_{c}^{\left(\mathrm{GA}\right)}$. However, the mutual relation between SGA and VMC results cannot be determined *a priori*, as the former provides better optimization of $|\psi_{0}\rangle$, whereas the latter puts more emphasis on the accurate calculation of averages in Equation (13). Looking at the results presented in Figure 5, we may conclude that $U_{c}^{\left(\mathrm{GWF}\right)}≳U_{c}^{\left(\mathrm{SGA}\right)}$, finding SGA as slightly less accurate, but promising (due to much lower computational costs) counterpart to VMC.

The VMC results concerning $U_{c}$ can be rationalized within a power law, with least-square fitted parameters, as follows

(23) $$\frac{t_{y}}{t_{x}}=\left(0.0269\pm 0.0014\right)\times {\left(\frac{U_{c}}{t_{x}}\right)}^{2.93\pm 0.05}.$$

(Here, a single standard deviation is given for each parameter.) The line given by Equation (23) [dashed-dotted], surrounded by the area [yellow] marking the statistical uncertainty, is further regarded as a border between semimetallic (SM) and Mott-insulating (MI) regions in the phase diagram. The former is further divided by marking the correlated-semimetal range (CSM), an appearance of which can be attributed to the fact that the HF approximation no longer produces a correct paramagnetic solution (m=0). Such a computation-oriented notion cannot be regarded as a thermodynamic phase *per se*; however, prominent effects of electron correlations, i.e., the band narrowing and the reduction in double occupancies are gradually amplified when the interaction is increased. These effects are further discussed in the next subsection, where we describe the behavior of measurable quantities when passing the CSM range and approaching the metal-insulator boundary.

Three of the methods (HF, GA, and GWF) indicate $U_{c}\to 0$ for $t_{y}/t_{x}\to 0$, coinciding with the exact solution for the Hubbard chain [3,4], giving an insulating phase at arbitrarily small U>0. In contrast, CPA and NGA produce $U_{c}>0$ in such a limit, showing that these are inapplicable in the limit of weakly-coupled chains, despite producing a reasonable results in the isotropic case. (In particular, when comparing to the value of $U_{c}/t_{0}=10$ given by DMFT [67].)

Two striking features of the data shown in Figure 5 are that most of the VMC datapoints do not match the GA line within the errorbars, but—on the other hand—the points for $t_{y}/t_{x}<0.8$ match the CPA results surprisingly close. The above may indicate a role of finite-size effects in VMC simulations (notice that both GA and CPA solutions correspond to the N→∞ limit). By manipulating the system sizes used for HF and GA calculations we found that shrinking to $N_{x}=N_{y}=10$ usually produces $U_{c}/t_{x}$ enlarged by 0.1 (HF) or 0.2 (GA) compared to the large-system limit. Therefore, one could roughly estimate $U_{c}^{\left(\mathrm{GWF}\right)}/t_{x}$ to be reduced by 0.2–0.3 when enlarging the system for $t_{y}\approx t_{x}$. This quantity is comparable but smaller than the deviation from the ‘exact’ QMC result of Ref. [18], namely $U_{c}^{\left(\mathrm{QMC}\right)}-U_{c}^{\left(\mathrm{GWF}\right)}\approx 0.4\;t_{x}$, suggesting that, in search for more accurate VMC results, one should first include additional variational parameters (such as Jastrow factors [68,69]), while the role of system size is rather secondary.

Moreover, in Figure 5 (bottom panel), we depict the trajectories followed by a sheet of graphene subjected to a strain in the armchair direction ($\varepsilon_{y}>0$) and allowed to relax in the perpendicular (i.e., zigzag) direction. The hopping matrix elements in the Hamiltonian (1) are parametrized according to [48,70,71]

(24) $$t_{ij}=-t_{0}\left(1-\beta \frac{\delta d_{ij}}{d_{0}}\right),$$

where β=2 (red solid line; open symbols) or β=3 (blue solid line; closed symbols) is the dimensionless electron–phonon coupling parameter. The bond-length variations ($\delta d_{ij}$) are adjusted to minimize the ground-state energy for an auxiliary Su–Schrieffer–Heeger model. (For more details, see Appendix B). The effective Hubbard repulsion is approximated as

(25) $$U_{\mathrm{eff}}=U-V_{01}{\langle \frac{d_{0}}{d_{ij}}\rangle }_{j(i)},$$

with the coefficients $U=3.63\;t_{0}$, and $V_{01}=2.03\;t_{0}$ taken from Ref. [23], and ${\langle \cdots \rangle }_{j(i)}$ denoting the average over three nearest neighbors *j* of the site *i*. (Due to our suppositions on the symmetry, Equation (25) produces same value for all sites.)

Depending on the electron–phonon coupling β, we find the strain of $\varepsilon_{y}=0.25$ (for β=2) or $\varepsilon_{y}=0.20$ (for β=3) is necessary to approach $U_{c}^{\left(\mathrm{HF}\right)}$, being a conventional border of the CSM range. These values are comparable with the maximal strain of ≈0.20 reported in experiments. The phase of the Mott Insulator seems inaccessible by applying mechanical strains to graphene, although modification of the equilibrium $U/t_{0}$ ratio due to the substrate effect may possibly enhance the interaction effects. Below, we discuss the effects of electron correlations which should also be visible in CSM (or even SM) phase.

### 3.2. Effects of Strain on Measurable Quantities

**Figure 6:**
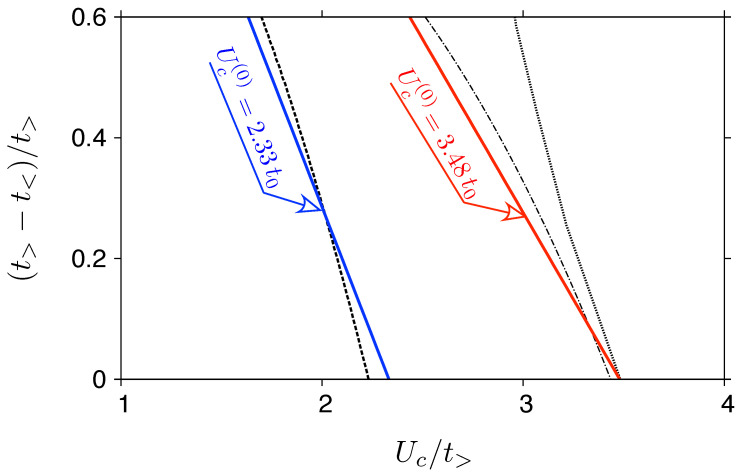
The evolution of critical Hubbard interaction with armchair strain strain ($t_{y}⩽t_{x}$), approximated by Equation (26) [solid lines] for two values of $U_{c}^{\left(0\right)}$ (specified on the plot) adjusted to match the zero-strain results obtained from HF and GWF methods. The remaining lines are the same as in Figure 5.

Earlier in this paper, we point out that a model assuming a linear density of states, Equation (11), parametrized by the Fermi velocity a zero energy, gives the critical Hubbard interaction $U_{c}^{\left(X\right)}$ that differs by only 5% from the values obtained using the actual density of states in the absence of strain, for the two methods, i.e., X= HF and X= GA. It is reasonable to expect that for strains introducing anisotropy of the Fermi velocity [72,73] the value of $U_{c}$ will be affected predominantly via a change of the cut-off energy Λ, related to the Fermi velocity. For $t_{y}⩽t_{x}$ and strains limited to experimentally-accessible values, one can set $\Lambda \propto \sqrt{t_{x}t_{y}}$, leading to

(26) $$U_{c}\approx U_{c}^{\left(0\right)}\left(1-\frac{1}{2}\delta_{t}\right),\;\;\;\;\delta_{t}=\frac{t_{x}-t_{y}}{t_{x}},$$

where $U_{c}^{\left(0\right)}$ a zero-strain value. Substituting the values of $U_{c}^{\left(\mathrm{HF}\right)}$ and $U_{c}^{\left(\mathrm{GWF}\right)}$ for $t_{y}=t_{x}$, we find (in Figure 6) that the evolution of $U_{c}/t_{x}$ with increasing strain is approximated by Equation (26) quite well for both HF and GWF methods (similar agreement is observed for GA results, omitted in Figure 6), but not for CPA, which predicts much weaker effects of strain.

**Figure 7:**
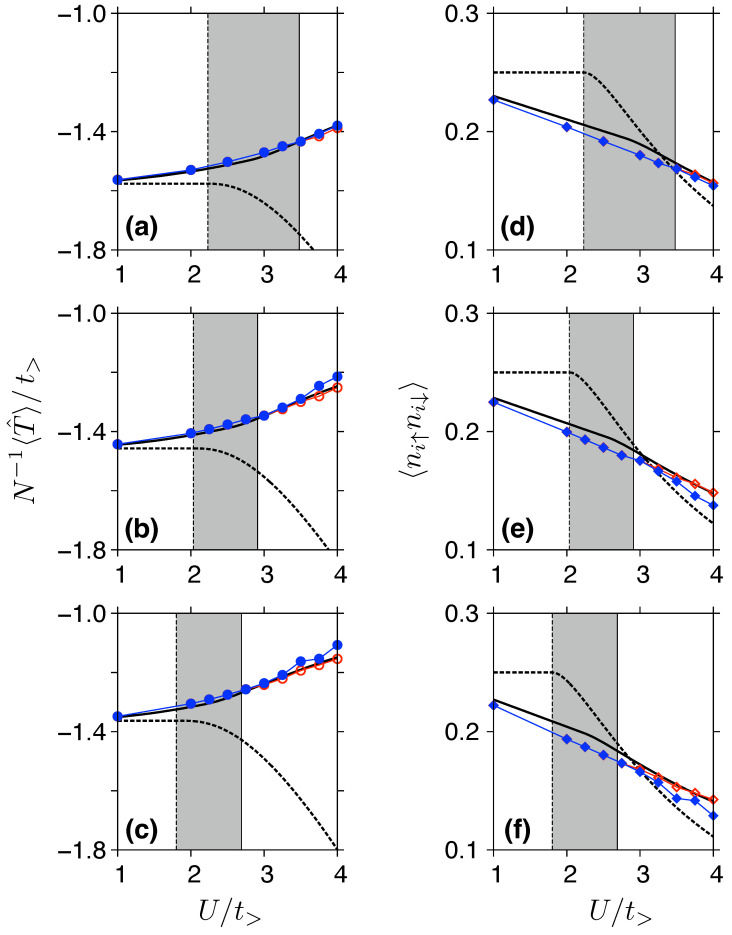
(**a**–**c**) Average kinetic energy per site and (**d**–**f**) average double occupancy displayed as functions of the Hubbard repulsion *U* for $t_{y}/t_{x}=1$ (top), $t_{y}/t_{x}=0.75$ (middle), and $t_{y}/t_{x}=0.5$ (bottom). Thick dashed line marks the Hartree–Fock results, thick solid line represents the Gutzwiller Approximation. Datapoints depict the VMC results for GWF with m=0 (red open symbols) and optimized *m* (blue closed symbols); thin lines are guide for the eye only. Shaded area marks the correlated semimetallic phase, bounded by $U_{c}^{\left(\mathrm{HF}\right)}$ and $U_{c}^{\left(\mathrm{GWF}\right)}$. (For the numerical values, see Table 1).

Our results (in particular, a systematic shrinking of the SM phase, as well as the CSM range, with increasing strain) suggest that some measurable signatures of electron correlations should also be visible in the strained system for $U<U_{c}$. This expectation is further supported by the data presented in Figure 7, where we display the average kinetic energy per site (quantifying the band narrowing) and the average double occupancy as functions of *U*. This time, the GWF results obtained from VMC simulations do not differ significantly from GA results (see datapoints and black solid lines, respectively), while HF (dashed lines) predict qualitatively different behavior, particularly for $\langle \hat{T}\rangle$ displayed versus *U* in Figure 7a–c, but the differences between HF and Gutzwiller-based techniques are also apparent for $\langle n_{i↑}n_{i↓}\rangle$ (see remaining panels in Figure 7).

For $\delta_{t}=0.5$, see Figure 7c,f, corresponding to the strain of $\varepsilon_{y}=0.22$ for β=3, and in the interval of $U/t_{x}=1.5÷2$ being relevant for graphene, the values of $\langle \hat{T}\rangle$ obtained from GWF or GA are reduced by more than 20% comparing the $\delta_{t}=0$ situation; see Figure 7a. (Notice that the above-mention reduction includes the change of $t_{x}$, used as an energy unit in Figure 7; the details are given in Appendix B.) Moreover, in the intermediate case, $\delta_{t}=0.25$, a 10% reduction is noticed, see Figure 7b. For $\langle n_{i↑}n_{i↓}\rangle$, the effect of strain is less pronounced, but we still have an approximately 10% reduction for the $\delta_{t}=0.5$ case [see Figure 7f] compared to the $\delta_{t}=0$ case [Figure 7d], following from both GWF or GA methods for the interval of $U/t_{x}=1.5÷2$.

## 4. Concluding Remarks

We have investigated the mutual effect of electron–electron interaction, modeled by a Hubbard term in the second-quantized Hamiltonian, and geometric strains applied to a half-filled honeycomb lattice, quantified (at a first step) via two arbitrary values of the nearest-neighbor hopping integrals: one for bonds inside zigzag lines parallel to a selected direction, and the other for remaining bonds. Related problems were widely studied in the existing literature [24,66,72,73]; therefore, our attention has focused on the case when strain is applied in the armchair direction (i.e., the hopping integrals connecting different zigzag lines are suppressed). In such a case, the energy spectrum for the noninteracting system remains gapless for arbitrary high strains, since the Dirac cones do not merge. In turn, the semimetal-insulator transition may occur only due to interactions. Moreover, the system gradually evolves, with increasing strain, towards a collection of weakly-coupled Hubbard chains, allowing us to expect a considerable reduction in the critical Hubbard repulsion.

Several computational methods are compared, finding that the Hartree–Fock (HF) approximation, Gutzwiller Approximation (GA), and Gutzwiller Wave Function (GWF) treated within Variational Monte Carlo simulations, all predict qualitatively-similar shrinking of the semimetallic phase with increasing strain. Two remaining methods, the Coherent Potential Approximation (CPA) and so-called Neél-state GA, produce slightly different shapes of the semimetal-insulator boundary, but in the CPA case, the results are numerically close to these obtained from GWF (provided that the strain is weak or moderate).

The phase diagram for the parametrized model is supplemented with calculations of trajectories, followed by monolayer graphene strained in the armchair direction and allowed to relax along the perpendicular (i.e., zigzag) direction. These calculations were performed employing modified Su–Schrieffer–Heeger Hamiltonian, including the harmonic terms for bonds and angles (with the parameters fixed to reproduce elastics properties for in-plane small deformations), and the term describing the coupling between electrons and the lattice, with dimensionless parameter varied between the possible values.

Albeit the critical and the actual Hubbard repulsion approach each other with increasing strain, we find that the semimetal-Mott insulator transition point cannot be achieved in monolayer graphene subjected to non-destructive deformations. Instead, one can observe the effects of electron correlations, such as the bandwidth renormalization or the reduction in double occupancies, which are well-pronounced (and affected by an applied strain) much before the transition. The above-mentioned effects will probably also be relevant in novel two-dimensional materials predicted to sustain geometric deformations up to about 30% without structural demages [74,75]. When looking for experimental realization of the Mott insulator on a honeycomb lattice, one should rather focus on artificial graphene-like systems [36,37,38,39].

What is more, we have shown that a basic version of the Gutzwiller Approximation already captures crucial correlation effects in (strained) graphene, allowing one to expect that proper generalizations of this method, such as the diagrammatic expansion [8,60,62], may lead to the development of versatile computational tools for studying graphene and related Dirac systems, providing low- (or moderate-) cost counterparts for the Quantum Monte Carlo methods.

## Figures and Tables

**Table 1:** Critical values of the Hubbard repulsion $U_{c}^{\left(\mathrm{GWF}\right)}$ obtained from VMC simulations (with standard deviations for the last digit specified in parentheses) compared with the upper ($U_{c}^{\left(\mathrm{GA}\right)}$) and the upper ($U_{c}^{\left(\mathrm{NGA}\right)}$) bound following from the Gutzwiller Approximation (GA) and the Néel-state Gutzwiller Approximation (NGA). The results obtained from the Statistically-consistent Gutzwiller Approximation (SGA) are also given. The system size is defined by $N_{x}=N_{y}=10$ for VMC simulations; the remaining results correspond to the limit of $N_{x}=N_{y}\to \infty$.

$\;\;t_{y}/t_{x}\;\;$	$\;U_{c}^{\left(\mathrm{GWF}\right)}\;/t_{x}\;$	$\;U_{c}^{\left(\mathrm{GA}\right)}\;/t_{x}\;$	$\;U_{c}^{\left(\mathrm{SGA}\right)}\;/t_{x}\;$	$\;U_{c}^{\left(\mathrm{NGA}\right)}\;/t_{x}\;$
1.00	3.48(1)	2.804	3.12	5.281
0.75	2.91(1)	2.550	2.83	4.871
0.50	2.69(3)	2.241	2.47	4.508
0.25	2.24(1)	1.830	1.98	4.199

**Figure A1:**
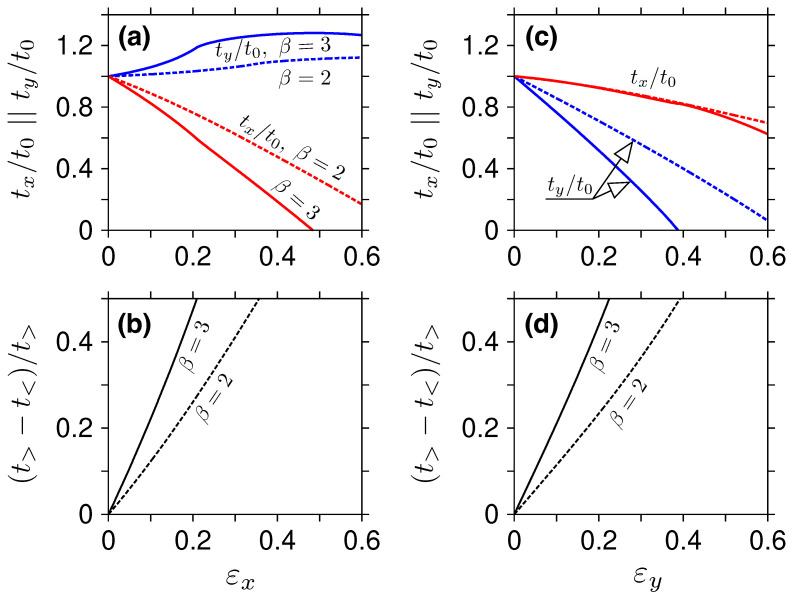
The hopping-matrix elements [see Equations (2) and (24) in the main text] as functions of the strain $\varepsilon_{x}>0$ (**a**,**b**) or $\varepsilon_{y}>0$ (**c**,**d**). The electron–phonon coupling is fixed at β=2 (dashed lines in all plots) or β=3 (solid lines).

## Data Availability

Datafiles will be provided upon reasonable request.

## Appendix A. Coherent Potential Approximation

The CPA method [16,17] employs the alloy-analogue approach, in which the many-body Hamiltonian (1), supplemented with the chemical potential term $-\mu \hat{N}$, is approximated by

(A1) $$\begin{array}{cc} H-\mu \hat{N} & \overset{\mathrm{CPA}}{=}\sum_{i\in A,s}E_{As}n_{is}+\sum_{j\in B,s}E_{Bs}n_{is}\\ \; & +\sum_{\langle ij\rangle ,s}t_{ij}\left(c_{is}^{†}c_{js}+\mathrm{H.c.}\right), \end{array}$$

where $\hat{N}=\sum_{is}n_{is}$ is the particle number operator, and the random potential energy

(A2) $$E_{\alpha s}=\left\{\begin{array}{cc} -\mu +U & \mathrm{with}\;\mathrm{probability}\;\;n_{\alpha \bar{s}},\\ -\mu & \mathrm{with}\;\mathrm{probability}\;\;1-n_{\alpha \bar{s}}. \end{array}\right.$$

Here, α=A,B is the sublattice index, $\bar{s}$ denotes the spin opposite to *s*, and $n_{\alpha \bar{s}}$ is the average occupation for spin $\bar{s}$ in the sublattice α. The half-filling corresponds to μ=−U/2.

The Green function for a single-particle Hamiltonian defined by Equations (A1) and (A2) needs to be averaged over all possible configurations of the random potential energies. Within the CPA, energies $E_{\alpha s}$ are approximated by self-energies $\Sigma_{\alpha s}\left(\omega \right)$, same for all atoms belonging to one sublattice. In turn, the Green function can be determined by solving the following system of self-consistent equations

$$\begin{array}{cc} G_{\alpha s}\left(\omega \right) & =\frac{F_{\alpha s}\left(\omega \right)\left(1-n_{\alpha \bar{s}}\right)}{1+F_{\alpha s}\left(\omega \right)\left[\Sigma_{\alpha s}\left(\omega \right)+\mu \right]} \end{array}$$

(A3) $$\begin{array}{cc} \; & +\frac{F_{\alpha s}\left(\omega \right)\;n_{\alpha \bar{s}}}{1+F_{\alpha s}\left(\omega \right)\left[\Sigma_{\alpha s}\left(\omega \right)+\mu -U\right]}, \end{array}$$

(A4) $$\begin{array}{cc} G_{\alpha s}\left(\omega \right) & =F_{\alpha s}\left(\omega \right), \end{array}$$

where $F_{\alpha s}\left(\omega \right)$ is the local Green function for the sublattice α,

(A5) $$F_{\alpha s}\left(\omega \right)=\xi_{\bar{\alpha }s}\int \frac{dE\;\rho (E)}{\xi_{As}\xi_{Bs}-E^{2}},$$

with $\xi_{\alpha s}=\omega +i\eta -\Sigma_{\alpha s}\left(\omega \right)$ and ρ(E) the density of states defined by Equation (9). Here, η>0 is a small real number (typically, we took $\eta /t_{x}=10^{-3}$).

Equations (A3) and (A4) can be solved iteratively via

(A6) $$\Sigma_{\alpha s}\left(\omega \right)=\Sigma_{\alpha s}\left(\omega \right)+\frac{1}{F_{\alpha s}\left(\omega \right)}-\frac{1}{G_{\alpha s}\left(\omega \right)}.$$

For simplicity, we limit our discussion to the paramagnetic solution, $n_{\alpha s}=n_{\alpha \bar{s}}=1/2$ for α=A,B. The multiparticle density of states is given (up to a normalization) by the imaginary part of $-G_{\alpha s}\left(\omega \right)$. This density of states, discussed as a function of *U* at a fixed ω=0, allows one to estimate the critical Hubbard repulsion $U_{c}^{\left(\mathrm{CPA}\right)}$, corresponding to the semimetal-insulator transition. Namely, $U_{c}^{\left(\mathrm{CPA}\right)}$ can be identified as the point when a nonzero −ImGα,s(0) rapidly drops to zero with increasing *U*; see Ref. [16].

## Appendix B. Su–Schrieffer–Heeger Model for Graphene

Below, we put forward a modified Su–Schrieffer–Heeger (SSH) model for tight-binding electrons adiabatically coupled to acoustic phonons [48,70,71], allowing one to map physical strains, applied to graphene, onto the Hamiltonian (1). For this purpose, we consider

(A7) $$\begin{array}{cc} H_{\mathrm{SSH}} & =-t_{0}\sum_{\langle ij\rangle ,s}\left(1-\beta \frac{\delta d_{ij}}{d_{0}}\right)\left(c_{i,s}^{†}c_{j,s}+\mathrm{H.c.}\right)\\ \; & +\frac{1}{2}K_{d}\sum_{\langle ij\rangle }{\left(d_{ij}\;-\;{\tilde{d}}_{0}\right)}^{2}+\frac{1}{2}K_{\theta }\;\sum_{j,∡(j)}\;{\left(\theta_{∡(j)}\;-\;\theta_{0}\right)}^{2}. \end{array}$$

Here, $t_{0}=2.7\;$ eV is the equilibrium nearest-neighbor hopping integral for π electrons in monolayer graphene, $\beta =-{\left.\partial \ln t_{ij}/\partial \ln d_{ij}\right|}_{d_{ij}=d_{0}}$ is the dimensionless parameter quantifying the electron–phonon coupling (later, we perform the main calculations for β=2 and β=3), and $\delta d_{ij}=d_{ij}-d_{0}$ is the bond-length change, calculated with respect to the equilibrium length of $d_{0}=a/\sqrt{3}=0.142\;$ nm). The second and third term in $H_{\mathrm{SSH}}$ represent potential energy describing the covalent bonds [49], with ∡(j) denoting the angles having a common vertex at a given lattice site *j* (see Figure 1).

The parameters $K_{d}=40.67\;$ eV/Å${}^{2}$, $K_{\theta }=5.46\;$ eV/rad ${}^{2}$, and $\theta_{0}=\pi /3$ are adjusted to restore the in-plane elastic coefficients of bulk graphene in the β=0 case [49]. For β≠0, a correction to the effective potential energy per bond $\left(2/3\right){\left.\partial^{2}\epsilon_{0}/\partial d_{ij}^{2}\right|}_{\left\{d_{ij}=d_{0}\right\}}=0$, provided that the equilibrium bond length is unaffected. To guarantee the last condition, we introduce the effective (β-dependent) equilibrium length

(A8) $${\tilde{d}}_{0}\left(\beta \right)=d_{0}+\frac{2\beta \epsilon_{0}}{3K_{d}d_{0}}\approx d_{0}\left(1+0.03455\;\beta \right),$$

where we have substituted the equilibrium kinetic energy per site $\epsilon_{0}=-1.57460\;t_{0}$. (Notice that a standard constraint to the SSH model, $\sum_{\langle ij\rangle }d_{ij}=\;$ const., is irrelevant when studying graphene with a global strain.)

The next step is the optimization of the ground-state energy,

(A9) $$E_{G}^{\left(\mathrm{SSH}\right)}\left(\left\{R_{j}\right\}\right)=\langle H_{\mathrm{SSH}}\rangle ,$$

with respect to in-plane atomic positions $\left\{R_{j}\right\}$. In this paper, we limit the discussion to atomic arrangements preserving the bipartite structure of the lattice and the two mirror symmetries. For a fixed strain in the selected direction, $\varepsilon_{>}=\max\left(\varepsilon_{x},\varepsilon_{y}\right)$, there are two parameters left to be optimized: the elongation in the perpendicular direction, $\varepsilon_{<}=\min\left(\varepsilon_{x},\varepsilon_{y}\right)$, and the length $d_{y}$ of the bonds parallel to *y*-axis. The length of the remaining bonds, belonging to the zigzag line, is given by

(A10) $$d_{x}=d_{0}{\left[\frac{3}{4}{\left(1+\varepsilon_{x}\right)}^{2}+\frac{9}{4}{\left(1+\varepsilon_{y}-\frac{2d_{y}}{3d_{0}}\right)}^{2}\right]}^{1/2}.$$

Numerical values of the hopping matrix elements, following from the optimization procedure for β=2, 3, and the two directions of strain are displayed in Figure A1.

In principle, the optimization scheme similar to the above can also be constructed for the Hamiltonian containing both lattice degrees of freedom and the Hubbard repulsion, $H_{\mathrm{SSH}}+U\hat{D}$. For instance, if $U<U_{c}$ and the paramagnetic phase can be assumed, one can refer directly to Equation (19), and setup a self-consistent procedure sharing the main features of the EDABI method [51], but not limited to small systems. However, the term $\propto U^{2}$ in Equation (19), introducing the coupling between lattice and electron correlations is preceded by a small prefactor and thus the corrections to atomic arrangements due to U>0 being insignificant (notice that the Hubbard repulsion for a monolayer in equilibrium is $U_{\mathrm{eff}}\approx 1.6\;t_{0}$ [23]). For these reasons, we decided not to pursue this direction here, limiting our presentation to the data obtained directly via minimizing $E_{G}^{\left(\mathrm{SSH}\right)}\left(\left\{R_{j}\right\}\right)$ in Equation (A9), leaving the geometric parameters and the correlation effects decoupled.
